# Cross shareholders and corporate cash holdings: Empirical evidence from Chinese listed companies

**DOI:** 10.1371/journal.pone.0338657

**Published:** 2026-10-26

**Authors:** Yili He, Xihe Liu

**Affiliations:** 1 School of Advanced Accounting and Auditing, Shanghai University of Finance and Economics, Shanghai, China; 2 School of Economics, Shanghai University, Shanghai, China; IU Internationale Hochschule GmbH, GERMANY

## Abstract

Cross shareholders have become a common phenomenon in capital markets, which increasingly draw the attention of scholars. This paper examines the effect of cross shareholders on corporate cash holdings in Chinese capital market and finds cross shareholders increase corporate cash holdings. Further mechanism analyses find suggestive evidence that this increase is correlated with reductions in overinvestment and improvements in total asset turnover. Additional results show the effect of cross shareholders has heterogeneous significance given the conditions of different external environment and firm characteristics. Moreover, cross shareholders reinforce the positive effect of cash holdings on firm productivity. Overall, this paper’s findings provide insight for understanding the role of cross shareholders in corporate cash holding decision in China.

## 1 Introduction

Cross shareholders, generally defined as shareholders holding shares in two or more companies within the same industry, have become a common phenomenon in capital markets. According to statistics, approximately one in five companies in the United States are connected because of BlackRock [1]. Similarly, in Europe, back in 1999, the aggregate market capitalization of companies with cross shareholders is accounted for 17% of the total market capitalization [2]. Although Chinese capital market is still in relatively early stages, cross shareholders are becoming increasingly common. Based on the data in this paper, approximately 14.3% of the samples involve cross shareholders.

Cross shareholders hold a large proportion of shares and have industry ties, so they are able to exert significant influence over the companies. Currently, a growing number of scholars begin to study cross shareholders. However, there remains controversy regarding the impact of cross shareholders on corporate governance. One category of existing research, adopting a corporate governance perspective, argues that cross shareholders can play a synergistic governance role within companies. However, the other side, adopting the perspective of Rational Economic Man Hypothesis, argues that that cross shareholders, for the purpose of maximizing profits, will impede competition within the industry and encourage collusion among their portfolio companies to pursue private gains [3,4].

Cross shareholder is essentially a form of horizontal merger with partial ownership. However, because of the debate regarding the role of cross shareholders, they have not yet been formally included within the regulatory scope by regulatory authorities. In light of this, it is crucial to clarify the role of cross shareholders.

Based on these two research perspectives, current research has linked cross shareholders to outcomes such as M&A transactions [5], corporate social responsibility [6], cost of equity capital [7], related party transactions [8], corporate innovation [9], tone of key audit matters [10], thereby enriching the understanding of cross shareholders’ economic impact. Regretfully, research on the relationship between cross shareholders and corporate cash holdings remains relatively scarce, and both the implications and underlying mechanisms require further investigation.

Cash holding is an important financial decision for companies. While cash offers high liquidity to meet daily operational needs, it is also vulnerable to misappropriation, potentially causing losses to the company. Therefore, maintaining a reasonable cash balance is crucial. Current studies on cash holdings have explored some equity factors such as large shareholder ownership and institutional ownership. However, these studies primarily focus on the influence of individual shareholders, neglecting the impact of shareholder networks such as cross shareholders.

This paper contributes to the literature in several important ways. First, existing research has primarily focused on institutional cross ownership [3,5,7–14]. Institutional investors are the dominant force in Western capital markets. However, as an emerging economy, Chinese capital market is still developing. Beyond institutional investors, individual investors also constitute a significant segment of the market. This paper examines all types of cross shareholders in China, including both institutional investors and individual investors, thereby helping to broaden the scope of research in cross shareholders. Second, Early research on cross shareholders focused on industrial competition [3,4]; later studies gradually expanded to the corporate governance area [8–10,12–14]. However, to date, no research has examined the impact of cross shareholders on corporate cash holdings. This study provides empirical evidence to comprehensively understand the impact of cross shareholders on cash holdings, clarifying the consequences and mechanisms of this influence. It enriches the heterogeneous conditions under which cross shareholders affect cash holdings and expands the associated economic outcomes. Thirdly, this paper enriches the understanding of cross shareholders’ impact outcomes, providing new evidence for their role in corporate governance. Fourth, it expands the theoretical boundaries of factors influencing cash holdings, demonstrating that shareholder networks are also significant determinants of cash holdings.

## 2 Literature review

### 2.1 Cross shareholders

Current research on cross shareholders primarily falls into two camps: one view holds that cross shareholders serve as a synergistic governance role in corporate operation, while the other argues that it leads to anti-competitive collusion.

From the perspective of synergistic governance, since cross shareholders hold shares in multiple companies within the same industry, they can leverage their informational advantages to reduce information asymmetry both within industries and within companies. At the same time, they can oversee and supervise management, thereby curbing opportunistic behavior from management. Specifically, the informational advantage of cross shareholders is primarily reflected in their extensive industry experience, information and resources, which can improve the firm’s information environment, reduce information asymmetry, alleviate financing constraints and promote corporate innovation [5,9,11,15]. The supervisory role of cross shareholders is reflected in their ability to better fulfill shareholder duties, such as expressing disagreement with the CEO during votes on proposals at shareholder meetings [13], removing incompetent CEOs [12], curbing CEOs’ manipulation of financial information [14], and constraining related party transactions [8]. By internalizing the externalities of corporate governance [13], cross shareholders have both the incentive and the ability to participate in corporate governance, thereby reducing agency problems.

From the perspective of anti-competitive collusion, rather than maximizing the profits of individual companies, cross shareholders are more concerned with maximizing the overall returns of their investment portfolios [10,16–18]. Intra-industry competition leads to increased costs, which reduces the returns of cross shareholders, thereby creating an incentive for them to promote collusive behavior among investee firms within the same industry.

### 2.2 Corporate cash holdings

According to the MM theory, in a perfectly efficient capital market, the costs of internal and external financing are identical; therefore, firms have no need to hold cash [19]. However, actually, due to transaction costs, information asymmetry, and agency problems, firms need to hold a certain amount of cash [20,21]. Early research summarized the motives for firms holding cash as transactional, precautionary, and speculative, while subsequent studies added tax avoidance and agency motives [22,23].

Chinese scholars argue that, as an emerging market, China faces high level of economic uncertainty and weak investor protection; so precautionary and agency motives are the primary factors influencing corporate cash holdings. The precautionary motive refers to firms holding large amounts of cash to hedge against future uncertainty and risk. Difficult financing conditions and high economic uncertainty both lead to an increase in precautionary cash holdings [23]. Under agency motives, management weighs the relative merits of current versus future consumption for their own benefit. If they favor the former, firms exhibit the high-cash-holding pattern proposed by the financial flexibility hypothesis; if they assign greater weight to the latter, firms exhibit the low-cash-holding pattern predicted by the dissipation hypothesis [24]. Chinese scholars find that due to the developing capital markets, weak legal protections for investors, and inefficient corporate governance mechanisms, the “dissipation hypothesis” is particularly prominent in China.

## 3 Hypothesis development

Previous studies indicate that cross shareholders may either facilitate synergistic governance or cause anti-competitive collusion. Meanwhile, in the context of Chinese capital markets, the primary motives influencing cash holdings are precautionary and agency motives, with the agency motive primarily manifesting as low-cash-holding pattern predicted by “dissipation hypothesis”. Based on these findings, this paper conducts a further analysis of the potential impact of cross shareholders on cash holdings.

### 3.1 Cross shareholders increase corporate cash holdings

Cross shareholders may play a synergistic governance role, thereby mitigating agency problems and increasing cash holdings. Cross shareholders can leverage their informational advantages to reduce information asymmetry and oversee management to curb opportunistic behavior of management. As mentioned earlier, under the background of developing capital market, weak investor protection and inefficient corporate governance mechanisms, the “dissipation hypothesis” proposed by the agency theory is more pronounced in China. Management, leveraging information advantage and authority, erodes the interests of the companies and smaller investors, particularly through wasteful expenditure of cash via mergers and acquisitions and other expansionary strategies. Therefore, the synergistic governance role of cross shareholders helps mitigate agency problems, reduce capital dissipation, and increase cash holdings.

### 3.2 Cross shareholders decrease corporate cash holdings

Cross shareholders may curb precautionary motive of cash holdings through synergistic governance or anti-competitive collusion, thereby leading to a reduction in cash holdings.

Driven by the precautionary motive, firms accumulate substantial cash reserves to hedge against future uncertainties and risks. The greater the information asymmetry, the more difficult it is for a company to raise funds from external sources, which means it has greater financing constraints. Consequently, the company has stronger incentive to hold cash as a precautionary measure. Cross shareholders can reduce the information gap between internal and external parties, thereby alleviating the company’s financing constraints and reducing its precautionary incentive to hold cash, which in turn lowers the company’s cash holding level.

On the other hand, beyond their governance role, cross shareholders may influence the precautionary motives for cash holdings by their anti-competitive collusion effect. To maximize the value of their whole investment portfolio, cross shareholders may reduce competition among companies in the same industry within their portfolio. Research has demonstrated that higher level of market competition correlates with increased corporate cash holdings [25,26]. As a result, cross shareholders reduce the amount of cash holdings which are originally retained to gain competitive advantage, thereby decreasing overall cash reserves.

Therefore, both of the two different roles cross shareholders play may influence precautionary motive for holding cash, thereby leading to a reduction in cash holdings. If the basic regression test reveals a decrease in cash holdings, further analysis is needed to clarify the mechanism through which cross shareholders exert their influence.

The preceding analysis indicates that, given the complexity of cross shareholder theory and cash holding theory, theoretical analysis aids in understanding potential causal relationships and mechanisms. However, cross shareholders wield the potential to yield both positive and negative impacts on corporate cash holdings, further empirical research being required to determine actual impact and underlying mechanisms. Therefore, this paper proposes the following hypotheses and will further examine the mechanisms through which cross shareholders influence corporate cash holdings as well as whether such influence ultimately enhances corporate operational efficiency.

H1a: Cross shareholders increase corporate cash holdings.
H1b: Cross shareholders decrease corporate cash holdings.

## 4 Research design

### 4.1 Sample

The original sample includes all Chinese A-shares listed companies from 2007 to 2024. The data has been acquired from the China Stock Market and Accounting Research (CSMAR) database. To improve data accuracy, we selected the research sample as follows. First, we restrict our sample to non-financial firms. Second, we exclude observations that are experiencing financial distress (designated as ST or *ST firms by a stock exchange). Third, we exclude observations with a debt-to-assets ratio greater than 1 or less than or equal to 0. Finally, we discard firm-year observations with missing data. The final sample includes 45213 firm-year observations. To mitigate the impact of extreme observations on our findings, all continuous variables are winsorized at the top and bottom 1%.

### 4.2 Model

By referring previous studies (e.g.[21,27–29],), we estimate the following model to explore the relationship between cross shareholders and corporate cash holdings:


$$\begin{array}{c} {Cash}_{i,t}=\beta_{\text{0}}+\beta_{\text{1}}{Cross}_{i,t}+\sum \beta_{j}{Controls}_{i,t}+\sum Industry+\sum Year+\varepsilon_{i,t} \end{array}$$ (1)

where i and t are firm and year indicators, respectively. The dependent variable is *Cash*. Consistent with prior literature (e.g.[21,30],), we use cash and cash equivalents divided by total assets to gauge the scale of corporate cash holdings.

*Cross* is the explanatory variable in Model (1) and represents for the quantity of cross shareholders. Because there are many zero values in the number of cross shareholders and the data exhibits right-skewness, this value cannot be used directly as a measure of *Cross*. As a result, by referring He and Huang [3] and Chen et al. [11], we measure the number of cross shareholders based on following steps. Specifically, for each quarter during 2007−2024 sample period, we extract Top 10 ownership and only keep the ownership which exceeds 5% of the outstanding shares. Using 5% ownership as a threshold is because shareholders hold more than 5% shares can exert significant influence over the company [31]. Shareholder is defined as a cross shareholder if he/she/it simultaneously holds more than one company in the same industry. Industry classification is based on the industry categorization of the Association of Listed Companies, with manufacturing subdivided into secondary codes and non-manufacturing enterprises subdivided into primary codes. We then calculate the annual average of the number of cross shareholders at the quarterly level, and take the natural logarithm of the sum of average data plus 1. The ln(1 + x) transformation can effectively mitigate data skewness while retaining information from the zero values.

Following previous studies (e.g.[21,23],), a set of control variable is added to Model 1 including firm size (*Size*), financial leverage (*Lev*), largest shareholder ownership (*Top1*), board size (*Board*), management pay (*TMTpay*) returns on assets (*ROA*), growth opportunity (*Growth*), quick ratio (*Quick*), financial constraint (*FC*), net working capital (*Nwc*), pay cash dividend or not (*Div*). Furthermore, we control for industry (*Industry*) and year (*Year*) fixed effects, respectively. The standard errors are adjusted for heteroscedasticity and clustered at the firm level. S1 Appendix provides detailed definitions for all variables.

## 5 Empirical results

### 5.1 Descriptive statistics

Descriptive statistics are reported in Table 1. The mean *Cash* is 0.159 and the standard deviation is 0.123; the maximum and minimum of *Cash* are 0.993 and 0, respectively. Apparently, there are significant differences in the cash holding of Chinese firms. The mean score for the independent variable *Cross* is 0.097, suggesting that there are about 0.102 cross shareholders on average in sample firm. The maximum and minimum of *Cross* is 1.609 and 0, respectively, suggesting that among sample firms the most has 4 cross shareholders, while the least has zero. The distributions of other variables are consistent with those reported in prior studies.

**Table 1:** Descriptive statistics.

Variable	N	Mean	SD	Min	p50	Max
*Cash*	45213	0.159	0.123	0	0.125	0.993
*Cross*	45213	0.097	0.249	0	0	1.609
*Size*	45213	22.24	1.284	19.83	22.05	26.27
*Lev*	45213	0.429	0.203	0.057	0.423	0.894
*Top1*	45213	0.338	0.148	0.084	0.315	0.738
*Board*	45213	2.121	0.201	1.609	2.197	2.708
*TMTpay*	45213	14.6	0.744	12.64	14.59	16.58
*ROA*	45213	0.033	0.063	−0.251	0.034	0.194
*Growth*	45213	0.145	0.373	−0.591	0.092	2.309
*Quick*	45213	1.915	2.148	0.181	1.219	13.64
*FC*	45213	3.852	0.273	3.151	3.846	4.556
*Nwc*	45213	−0.06	0.543	−2.987	0.047	1.078
*Div*	45213	0.715	0.451	0	1	1

Note: S1 Appendix lists detailed definitions of all variables. The same below.

Table 2 shows the results of Pearson correlation coefficient tests for the control variables. The correlation coefficients of most variables are less than 0.5. Furthermore, we conducted variance inflation factor (VIF) tests. All of the variance inflation factors are less than 5, indicating that there are no significant multi-collinearity issues in the model.

**Table 2:** Results of correlation coefficients for control variables.

	(1)	(2)	(3)	(4)	(5)	(6)	(7)	(8)	(9)	(10)	(11)
*Size*	1										
*Lev*	0.46***	1									
*Top1*	0.19***	0.04***	1								
*Board*	0.24***	0.15***	0.04***	1							
*TMTpay*	0.44***	0.03***	−0.05***	0.0100	1						
*ROA*	0.05***	−0.34***	0.16***	0.04***	0.15***	1					
*Growth*	0.03***	0.04***	0.03***	0.02***	0	0.26***	1				
*Quick*	−0.31***	−0.66***	−0.03***	−0.14***	0	0.20***	−0.05***	1			
*FC*	0.05***	0.05***	−0.16***	−0.05***	0.23***	−0.09***	−0.09***	−0.08***	1		
*Nwc*	−0.20***	−0.52***	−0.02***	−0.13***	0.07***	0.25***	0.03***	0.28***	−0.03***	1	
*Div*	0.15***	−0.22***	0.16***	0.05***	0.19***	0.51***	0.11***	0.13***	−0.07***	0.22***	1

Note: ***, ** and * indicate statistical significance at 1%, 5% and 10% levels respectively (two-tailed).

### 5.2 Baseline results

Table 3 presents the regression results for our baseline model. The coefficient of *Cross* is −0.002 but not significant when control variables are removed in Column (1). However, when control variables are added in Column (2), the coefficient of *Cross* is 0.014, positively significant at the 1% level. The results in two regressions indicate that omitted variable may exist in univariate regression, leading to biased results. The estimation in Column (2) means that, a one-standard-deviation increase in *Cross* is associated with an increase of 0.003 in *Cash*, approximately 3% of its standard deviation. The baseline results therefore support H1a, providing preliminary evidence that cross shareholders increase corporate cash holdings by mitigating agency problems. In contrast, we find no empirical support for H1b. A plausible explanation is that the corporate governance environment in China remains in a developmental stage, and agency problems continue to play a prominent role in corporate decision‑making. Under such circumstances, agency problems may exert a more pronounced influence on cash holdings than precautionary motive, making the former channel relatively more dominant. The subsequent mechanism tests will further empirically examine the specific pathways through which this effect operates.

**Table 3:** Baseline results.

Variable	(1)	(2)
	*Cash*	*Cash*
*Cross*	−0.002	0.014***
	(−0.47)	(3.57)
*Size*		−0.008***
		(−6.43)
*Lev*		−0.096***
		(−11.62)
*Top1*		0.039***
		(5.14)
*Board*		0.017***
		(3.15)
*TMTpay*		0.012***
		(6.46)
*ROA*		0.241***
		(13.57)
*Growth*		−0.001
		(−0.70)
*Quick*		0.022***
		(22.69)
*FC*		−0.008*
		(−1.76)
*Nwc*		−0.040***
		(−17.65)
*Div*		0.014***
		(7.4)
*Industry FE*	NO	YES
*Year FE*	NO	YES
Constant	0.131***	0.147***
	(13.6)	(4.34)
*N*	45213	45213
Adjusted R^2^	0.1	0.355

Note: Robust t-values adjusted by heteroscedastic and firm-level cluster standard errors are below the regression coefficients in parentheses, ***, ** and * indicate statistical significance at 1%, 5% and 10% levels respectively (two-tailed). The same below.

### 5.3 Robustness checks

#### 5.3.1 Instrumental variable analysis.

Cross shareholders do not randomly invest. It is plausible that cross shareholders prefer firms with high cash holdings, so the paper may suffer from the reverse causality problem. Instrumental variable is used to mitigate potential endogeneity concerns resulting from unobservable characteristics. Specifically, this paper includes instrumental variable *Cross_IV*, which denotes the inverse of the weighted average distance of each company from the two major financial centers, Shanghai and Shenzhen, multiplied by the company’s listing age (lagged by one period).

The amount of cross shareholders depends largely on potential investor resources. Shanghai and Shenzhen have well-established capital markets, offering abundant investor resources. As a result, companies located near these two financial centers tend to attract more cross shareholders. However, the proximity to Shanghai and Shenzhen does not necessarily imply a higher level of regional financial development. A case in point is Beijing, which, despite its greater geographic distance from Shanghai and Shenzhen, ranks among the most financially developed regions in China. Therefore, one cannot infer the degree of regional financial development solely from the distance to Shanghai and Shenzhen, let alone establish a direct link between this distance and corporate cash holdings.

Besides, to mitigate potential confounding effects from firm‑specific and regional factors, we have further controlled for firm listing age (*Age*), regional financial development (*Finance*) and province fixed effects in the instrumental variable analysis.

Table 4 reports the results of this analysis. Column (1) shows the results of the first-stage regression. *Cross_IV* is positively correlated with *Cross* at 1% significant level, which is in line with our expectations. We further test the validity of the instrumental variables by conducting the under-identification test and the weak identification test. Specifically, The Kleibergen-Paap rk LM statistic is 22.50 and the Kleibergen-Paap rk Wald F statistic is 23.05, which pass the under-identification test and the weak identification test, respectively. Column (2) shows the results of the second-stage regression. We find that the coefficient of *Cross* is 0.484, significant at 1% level. Notably, the IV estimate is larger than its OLS counterpart. This difference suggests that the OLS estimate is likely affected by endogeneity, and the IV approach effectively mitigates this issue by isolating exogenous variation, yielding a larger and more reliable estimate. The total findings indicate that IV approach effectively alleviates endogeneity concerns and delivers more robust results.

**Table 4:** Results of Instrumental variable analysis.

Variable	1^st^ Stage	2^nd^ Stage
	(1)	(2)
	*Cross*	*Cash*
*Cross_IV*	3.343***	
	(4.80)	
*Cross*		0.484***
		(4.06)
Kleibergen-Paap rk LM statistic	22.50***	
Kleibergen-Paap rk Wald F statistic	23.05***	
Controls	YES	YES
Industry FE	YES	YES
Year FE	YES	YES
Province FE	YES	YES
Constant	−0.705***	0.506***
	(−7.34)	(4.72)
*N*	45213	45213

#### 5.3.2 Heckman test.

To further mitigate self-selection bias, this paper employs the Heckman two-stage method. Specifically, we construct a Probit model to examine the relationship between firms’ characteristics in the previous period and the presence of cross shareholders (*Cross_Dummy*) in the current period. The specific model is as follows:


$$\begin{array}{c} {Cross\_Dummy}_{i,t}=\beta_{\text{0}}+\sum \beta_{j}{Lag\_Controls}_{i,t}+\sum Industry+\sum Year+\varepsilon_{i,t} \end{array}$$ (2)

*Cross_Dummy* is a dummy variable with value one if the firm-years observation has at least one cross shareholder and zero otherwise. *Lag_Controls* is a set of control variables lagged by one period, including firm size (*Size*), financial leverage (*Lev*), largest shareholder ownership (*Top1*), return on equity (*ROE*), growth opportunity (*Growth*), quick ratio (*Quick*), pay cash dividend or not (*Div*), as well as net profit margin (*Netprofit*). The reason for lagging control variables is that investors can only obtain financial and governance information about a company from its previous financial statements when buying or selling stocks. Based on Model (2), this paper constructs the Inverse Mills Ratio (*IMR*) to capture the impact of prior-period financial and corporate governance variables on the presence of cross shareholders (*Cross_Dummy*). This *IMR* is then included as a control variable in the baseline regression to correct for potential selection bias that might otherwise distort the study’s findings.

The results are shown in Table 5. The coefficient of *IMR* is significantly positive at 1% level, indicating that a distribution bias in the sample of cross shareholders does indeed exist; therefore, it is necessary to account for the estimation bias that may result from this sample self-selection. Furthermore, the coefficient of *Cross* remains significantly positive at the 5% level, consistent with the baseline results, indicating that the conclusions of this paper remain valid even after controlling for selection bias.

**Table 5:** Results of Heckman test.

Variable	*Cash*
*Cross*	0.012*
	(1.89)
*IMR*	0.108***
	(4.99)
Controls	YES
Industry FE	YES
Year FE	YES
*N*	39856

Note: The samples are less than ones in baseline regression because *IMR* is measured by a set of control variables lagged by one period.

#### 5.3.3 Entropy balancing technique.

Firms with cross shareholders and firms without cross shareholders have big difference. We use an entropy balancing technique to match the covariates across the firms with and without cross shareholders. This approach enables to achieve higher degree of covariate balance with fewer restrictive assumptions and without dropping any observations [32]. To assign scalar weights to all of the sample, the entropy balancing technique balances the covariate distributions of the treatment and control groups across their mean, variation and skewness. The results in Table 6 suggests that after matching there are no significant difference between the two groups. Table 7 reports the result for the matched sample based on entropy balancing. The coefficient of *Cross* is significantly positive at 5% level, supporting our main findings.

**Table 6:** Covariate balance of entropy balancing.

Variable	Treat			Control before balancing			Control after balancing		
	Mean	Var	Skew	Mean	Var	Skew	Mean	Var	Skew
*Size*	22.930	2.296	0.426	22.130	1.449	0.780	22.930	2.296	0.427
*Lev*	0.481	0.039	−0.102	0.420	0.041	0.210	0.481	0.039	−0.102
*Top1*	0.350	0.022	0.441	0.336	0.022	0.536	0.350	0.022	0.441
*Board*	2.183	0.042	−0.114	2.110	0.039	−0.345	2.183	0.042	−0.114
*TMTpay*	14.710	0.574	0.087	14.580	0.548	0.057	14.710	0.574	0.088
*ROA*	0.031	0.003	−1.179	0.033	0.004	−1.269	0.031	0.003	−1.179
*Growth*	0.140	0.133	2.920	0.146	0.140	2.540	0.140	0.133	2.920
*Quick*	1.539	3.248	3.963	1.978	4.812	3.046	1.539	3.248	3.963
*FC*	3.842	0.092	−0.180	3.853	0.072	0.120	3.842	0.092	−0.179
*Nwc*	−0.183	0.355	−2.114	−0.039	0.282	−2.479	−0.183	0.355	−2.114
*Div*	0.729	0.198	−1.030	0.713	0.205	−0.940	0.729	0.198	−1.030

**Table 7:** Regression results after entropy balancing.

Variable	(1)
	*Cash*
*Cross*	0.008**
	(2.15)
*Controls*	YES
Industry FE	YES
Year FE	YES
Constant	0.079*
	(1.72)
*N*	45213
Adjusted R^2^	0.369

#### 5.3.4 Alternative fixed effects.

Different areas may influence the cash holding level of firms, so province fixed effect (*Province*) is added to Model (1).

Additionally, time-varying industry characteristics such as cyclical change, industrial policy and monetary policy may have had differential effects on the association between cross shareholders and corporate cash holdings [15]. These factors may also influence the stock holding choices and portfolio rebalancing intentions of cross shareholders as well as the cash holding decision of the focal firm. Similarly, time-varying regional characteristics may have had differential effects on the association between cross shareholders and corporate cash holdings. Therefore, to reduce the potential bias caused by heterogeneous concerns for time-trend industries and regional effects, we also add *Industry*×*Year* and *Province*×*Year* interactive fixed effects into Model (1), respectively. Columns (1)-(3) in Table 8 show the results when control *Province* fixed effects, *Industry*×*Year* fixed effects and *Province*×*Year* fixed effects. The coefficient of *Cross* is positive and significant at 1% level, which supports our main findings.

**Table 8:** Results of alternative fixed effects.

Variable	(1)	(2)	(3)
	*Cash*	*Cash*	*Cash*
*Cross*	0.012***	0.012***	0.012***
	(2.96)	(3.01)	(3.04)
*Controls*	YES	YES	YES
Industry FE	YES	NO	YES
Year FE	YES	YES	YES
Province FE	YES	YES	NO
Industry× Year FE	NO	YES	NO
Province×Year FE	NO	NO	YES
Constant	0.155***	0.131***	0.159***
	(4.59)	(3.32)	(4.32)
*N*	45213	45213	45213
Adjusted R^2^	0.360	0.365	0.362

Note: In Column (1), this paper includes *Province* to control province fixed effect. In Column 2, this paper includes *Industry*×*Year* interactive fixed effects to control time-trend industries effect. In Column (3), this paper includes *Province*×*Year* interactive fixed effects to control time-trend regional effect.

#### 5.3.5 Alternative measures.

We then repeat the regression analyses using alternative metrics for both the dependent and independent variables. Specifically, referring previous studies [3,11], we remeasure cross shareholders with the dummy variable *Cross_Dummy* as well as *Cross_Share* which is the total ownership of all cross shareholders in certain firm in certain year. As reported in Panel A of Table 9, the coefficient of *Cross_Dummy* and *Cross_Share* are significantly positive at 1% level, consistent with our main results.

**Table 9:** Results of alternative measures.

Panel A:Alternative independent variables		
**Variable**	**(1)**	**(2)**
	***Cash***	***Cash***
*Cross_Dummy*	0.010***	
	(3.61)	
*Cross_Share*		0.025***
		(2.82)
Controls	YES	YES
Industry FE	YES	YES
Year FE	YES	YES
Constant	0.146***	0.145***
	(4.32)	(4.30)
*N*	45213	45213
Adjusted R^2^	0.355	0.355
Panel B:Alternative dependent variables		
Variable	(1)	(2)
	*Cash1*	*Cash2*
*Cross*	0.014***	0.025***
	(3.59)	(2.63)
Controls	YES	YES
Industry FE	YES	YES
Year FE	YES	YES
Constant	0.019	0.339**
	(0.57)	(2.12)
*N*	45213	45213
Adjusted R^2^	0.272	0.067

Note: Panel A presents the regression results of alternative independent variables. Panel B presents the regression results of alternative dependent variables.

Moreover, considering different industry has different cash holding level, we remeasure the cash holding level by using the industry-year adjusted proxy. Specifically, *Cash1* is generated by using *Cash* minus the industry-year mean of *Cash*. Besides, previous studies suggested another way to gauge the cash holding level [33], which is cash and cash equivalents divided by total assets excluding cash and cash equivalents (*Cash2*). Panel B of Table 9 presents the results. The coefficients of *Cross* are still positively significant no matter which dependent variable used, indicating that the paper’s findings are robust.

#### 5.3.6 Using different samples.

Referring previous studies, this paper further considers the influence of external event shock. Specifically, this paper considers the influence of the financial crisis in 2008, the domestic stock market crash in 2015 and the COVID-19 epidemic in 2020. Table 10 shows the results. Column (1) – (4) are the results when eliminating the influence of the financial crisis in 2008, the domestic stock market crash in 2015, the COVID-19 epidemic in 2020, and all the three years. The coefficients of *Cross* are positive and significant in all columns of Table 10, indicating that the baseline results are robust.

**Table 10:** Results of using different samples.

Variable	(1)	(2)	(3)	(4)
	*Cash*	*Cash*	*Cash*	*Cash*
*Cross*	0.014***	0.014***	0.013***	0.014***
	(3.62)	(3.63)	(3.40)	(3.52)
Controls	YES	YES	YES	YES
Industry FE	YES	YES	YES	YES
Year FE	YES	YES	YES	YES
Constant	0.148***	0.145***	0.148***	0.148***
	(4.35)	(4.28)	(4.37)	(4.34)
*N*	44116	42978	42013	38681
Adjusted R^2^	0.354	0.356	0.358	0.358

Note: In Column (1), (2) and (3), this paper eliminates the financial crisis in 2008, the domestic stock market crash in 2015 and the influence of the COVID-19 epidemic in 2020 respectively. This paper excludes the aforementioned three years in Column (4).

#### 5.3.7 Placebo test.

We also conduct a placebo test as the robustness test. This paper randomly assigns variable *Cross* into firm-year observations each time and run the regression. Such procedures are repeated 1000 times. Fig 1 shows the kernel density coefficient distribution of 1000 regressions, suggesting that the t-values are predominantly distributed around zero. Overall, the findings suggest the resilience of baseline results.

**Fig 1:**
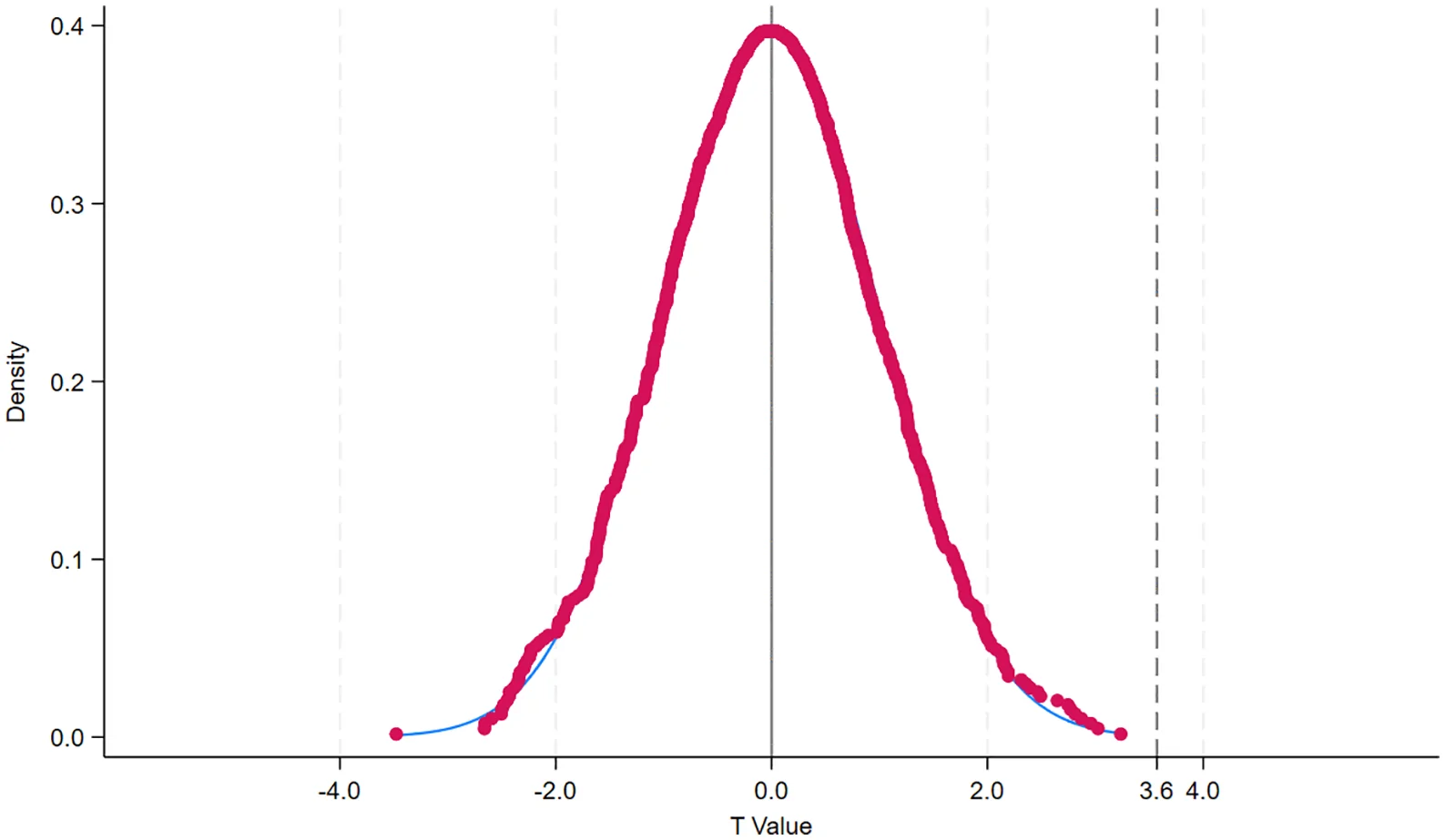
Placebo test. Note: This paper randomly assigns variable *Cross* into firm-year observations each time and run the regression. Such procedures are repeated 1000 times. This figure shows the kernel density coefficient distribution of 1000 regressions.

#### 5.3.8 Other robustness checks.

To enhance the robustness of our findings, we re‑estimated our main regression using both the Tobit model and the firm fixed effects model. The results are reported in S2 Appendix.

## 6 Further analysis

### 6.1 Mechanism analysis

Basic results show cross shareholders can improve the cash holding level of companies. As discussed in Section 3, cross shareholders can oversee management, thereby alleviating the agency problems. This paper further examines the impact of cross shareholders on agency problems from both the input and output perspectives. Specifically, we construct the following mediating models.


$$\begin{array}{c} {Overinv}_{i,t}/{ATO}_{i,t}=\beta_{\text{0}}+\beta_{\text{1}}{Cross}_{i,t}+\sum \beta_{j}{Controls}_{i,t}+\sum Industry+\sum Year+\varepsilon_{i,t} \end{array}$$ (3)

$$\begin{array}{c} {Cash}_{i,t}=\beta_{\text{0}}+\beta_{\text{1}}{Cross}_{i,t}+\beta_{\text{2}}{Overinv}_{i,t}/{ATO}_{i,t}+\sum \beta_{j}{Controls}_{i,t}+\sum Industry+\sum Year+\varepsilon_{i,t} \end{array}$$ (4)

In order to examine the impact of cross shareholders on agency problems from an input perspective, this paper constructs a proxy for overinvestment (*Overinv*) by referring the work of Biddle et al. [34]. In terms of output, this paper follows the research of Ang et al. [35] and builds a proxy for total asset turnover ratio (ATO). The higher the total asset turnover ratio, the less severe the agency problem. This variable can be used to measure the magnitude of the agency problem from an output perspective.

Table 11 Panel A and Panel B report the results. Column (1) in Panel A shows the coefficient of *Cross* is negatively significant at 1%. Column (2) in Panel A shows the coefficient of *Overinv* is negatively significant at 1%. Total results in Panel A shows cross shareholders are associated with a reduction in overinvestment, which offers suggestive evidence for one potential channel through which cross shareholders may influence corporate cash holdings. Column (1) in Panel B shows the coefficient of *Cross* is positively significant at 5%. Column (2) in Panel B shows the coefficient of *ATO* is positively significant at 1%. Total results in Panel B shows cross shareholders are correlated with a mitigation of agency problems. The mitigation in ineffective dissipation caused by agency problems leads to an increase in total asset turnover, suggesting a possible channel through which cross shareholders affect corporate cash holdings.

**Table 11:** Results of mechanism analysis.

Panel A: Overinvestment		
Variable	(1)	(2)
	*Overinv*	*Cash*
*Cross*	−0.005***	0.013***
	(−3.97)	(3.41)
*Overinv*		−0.159***
		(−11.07)
Controls	YES	YES
Industry FE	YES	YES
Year FE	YES	YES
Constant	0.086	0.160***
	(7.47)	(4.76)
*N*	45213	45213
Adjusted R^2^	0.083	0.358
Panel B: Total asset turnover ratio		
Variable	(1)	(2)
	*ATO*	*Cash*
*Cross*	0.036**	0.013***
	(1.97)	(3.38)
*ATO*		0.027***
		(9.49)
Controls	YES	YES
Industry FE	YES	YES
Year FE	YES	YES
Constant	0.042	0.146***
	(0.30)	(4.34)
*N*	45213	45213
Adjusted R^2^	0.340	0.361

### 6.2 Heterogeneity analysis

Given the potential heterogeneous effect of cross shareholders on corporate cash holdings across different scenarios, this paper intends to conduct an analysis from the perspective of external environment heterogeneity and firm characteristics heterogeneity.

#### 6.2.1 External environment.

The relationship between cross shareholders and corporate cash holdings is influenced by the external environment. Therefore, this paper selects economic policy uncertainty, which reflects macroeconomic conditions, and industry competition, which reflects mesoeconomic conditions, for further exploration.

As economic policy uncertainty increases, the complexity and dynamism of the external business environment also grow, thereby impacting corporate business decisions [36]. Study has shown when the uncertainty of economic policy is high, the external risk also increases [37], so it is difficult for management to improve corporate performance through their efforts. Moreover, economic policy uncertainty has weakened regulatory enforcement and increased opportunistic behavior by management. These can inadvertently prompt management to act in their own best interests, exacerbating agency problems within the company. In such circumstances, cross shareholders can play a more significant role. To verify the validity of this analysis, the study adds economic policy uncertainty (*Epu*) and the cross-term between economic policy uncertainty and cross shareholders (*Cross*×*Epu*) to the baseline regression to examine the moderating effect of economic uncertainty on the correlation between cross shareholders and corporate cash holdings. Economic policy uncertainty (*Epu*) is calculated according to the method used by Baker et al. [36]. This paper first calculates the monthly economic policy uncertainty index, then finds its annual average, and finally takes the natural logarithm of the annual average. The higher *Epu* index, the higher economic policy uncertainty.

By referring previous studies, industry competition creates external governance mechanisms for enterprises, which creates a favorable environment for governance mechanisms to perform effectively [38,39]. As the intensity of industry competition standardizes the governance environment, the effect of cross shareholders may be significant. Based on this prediction, the positive effect of cross shareholders on corporate cash holdings is more pronounced when industry competition is high. To validate this analysis, this study incorporates market competition intensity (*HHI*) and the interaction term between market competition intensity and cross shareholders (*Cross×HHI*) into the baseline regression to examine the moderating effect of industry competition intensity on the relationship between cross shareholders and corporate cash holdings. Market competition intensity (*HHI*) is measured using the Herfindahl-Hirschman Index, calculated as follows: first, the proportion of a single company's main business revenue relative to the total main business revenue of all companies in its industry is calculated; then sum the squared proportion by industry. A higher *HHI* value indicates less intense market competition.

In Table 12, regression results in Column (1) show that the coefficient of *Cross*×*Epu* is significantly positive at the 1% level, indicating that cross shareholders are more effective in exercising supervisory functions when economic policy uncertainty is high. Regression results in Column (2) show that the coefficient of *Cross×HHI* is significantly negative at the 1% level, indicating that cross shareholders are more effective in industries with higher levels of competition.

**Table 12:** Test results for heterogeneity of external environment.

Variable	(1)	(2)
	*Cash*	*Cash*
*Cross*	−0.009	0.024***
	(−1.53)	(4.43)
*Cross* × *Epu*	0.005***	
	(4.64)	
*Epu*	−0.005***	
	(−5.12)	
*Cross* × *HHI*		−0.113**
		(−2.53)
*HHI*		0.018
		(1.32)
Controls	YES	YES
Industry FE	YES	YES
Year FE	YES	YES
Constant	0.153***	0.142***
	(4.58)	(4.17)
*N*	45213	45213
Adjusted R^2^	0.356	0.355

#### 6.2.2 Firm characteristics.

Grossman and Hart [40] argue that when equity concentration is high, the interests of large shareholders tend to align with those of the company, thereby strengthening the motivation of large shareholders to participate in corporate management, reducing free riding, and ultimately improving performance. Shleifer and Vishny [41] also argue that due to the agency problem between shareholders and management, equity concentration enables large shareholders to effectively supervise and check management, thereby improving corporate performance. Therefore, this paper predicts that in companies with high equity concentration, the supervisory role of cross shareholders is not significant due to the substitution of large shareholders, while in companies with dispersed equity, the supervisory effect of cross shareholders is more pronounced. This study incorporates equity concentration (*Share_HHI*) and the interaction term between equity concentration and cross shareholders (*Cross*×*Share_HHI*) into the baseline regression. *Share_HHI* is calculated by squaring the shareholding ratios of the top 10 shareholders and summing the results. A higher *Share_HHI* value indicates greater equity concentration within the company.

Prior literature points out that analysts serve as the information channel of the capital markets; their attention enhances transparency in the capital markets and alleviates information asymmetry [42]. Analyst attention may substitute the governance role of cross shareholders. As a result, it is speculated that the synergistic governance role of cross shareholders will be more obvious in companies with less analyst attention. This study introduces analyst attention *(Analyst*) and the interaction term between analyst attention and cross shareholders (*Cross*×*Analyst*) into the baseline regression. Analyst attention is calculated by the number of analysts covering the same listed company in a year plus 1, and then taken the natural logarithm. A higher *Analyst* value indicates greater analyst attention on the company.

Table 13, column (1) regression results show that the coefficient of *Cross*×*Share_HHI* is significantly negative at the 1% level, indicating that when equity concentration is low, the role of cross shareholders is more prominent. Column (2) regression results show that the coefficient of *Cross*×*Analyst* is significantly negative at the 1% level, indicating that when a company has less analyst attention, cross shareholders are more effective in increasing corporate cash holdings.

**Table 13:** Test results for heterogeneity of firm characteristics.

Variable	(1)	(2)
	*Cash*	*Cash*
*Cross*	0.032***	0.027***
	(5.07)	(4.67)
*Cross* × *Share_HHI*	−0.104***	
	(−3.57)	
*Share_HHI*	−0.031	
	(−1.03)	
*Cross* × *Analyst*		−0.008***
		(−2.80)
*Analyst*		0.006***
		(5.76)
Controls	YES	YES
Industry FE	YES	YES
Year FE	YES	YES
Constant	0.137***	0.191***
	(4.04)	(5.46)
*N*	45213	45213
Adjusted R^2^	0.356	0.357

### 6.3 Economic consequence test

Previous sections have demonstrated that cross shareholders can increase corporate cash holdings through synergistic governance. Cash management is an essential part of sound corporate operations. Beyond their significant influence on corporate cash holdings, whether cross shareholders can impact operational efficiency by affecting cash holdings has become a further focus of our attention. This helps us understand the positive role cross shareholders play in the company as a whole. This paper employs total factor productivity (*TFP*) as the proxy for corporate operational efficiency. Regarding *TFP* estimation methods, prior research has demonstrated that the LP method effectively addresses endogeneity issues and sample selection bias. Therefore, this study calculates *TFP* through LP method. This paper constructs the following model:


$$\begin{array}{c} {TFP}_{i,t}=\beta_{\text{0}}+\beta_{\text{1}}{Cash}_{i,t}+\sum \beta_{j}{Controls}_{i,t}+\sum Industry+\sum Year+\varepsilon_{i,t} \end{array}$$ (5)

$$\begin{array}{c} {TFP}_{i,t}=\beta_{\text{0}}+\beta_{\text{1}}{Cash}_{i,t}+\beta_{\text{2}}{Cross}_{i,t}\times {Cash}_{i,t}+\beta_{\text{3}}{Cross}_{i,t}+\sum \beta_{j}{Controls}_{i,t}+\sum Industry+\sum Year+\varepsilon_{i,t} \end{array}$$ (6)

The coefficient of *Cash* in Model (5) examines the impact of cash holdings themselves on firm productivity. The coefficient of *Cross*×*Cash* in Model (6) assesses the effect of cross shareholders on the association between cash holdings and total firm productivity. Table 14, Column (1) shows that the coefficient of *Cash* is significantly positive, indicating that cash holdings contribute to enhancing firm productivity. Column (2) shows that the interaction term between *Cross* and *Cash* is significantly positive, indicating that cross shareholders reinforce the positive effect of cash holdings on firm productivity.

**Table 14:** Cash holding and firm productivity.

Variable	(1)	(2)
	*TFP*	*TFP*
*Cash*	0.881***	0.841***
	(16.98)	(16.17)
*Cross* × *Cash*		0.421**
		(2.43)
*Cross*		−0.008
		(−0.21)
Controls	YES	YES
Industry FE	YES	YES
Year FE	YES	YES
Constant	−7.003***	−6.944***
	(−34.80)	(−34.24)
*N*	45213	45213
Adjusted R^2^	0.767	0.768

## 7 Conclusion

This study investigates the effect of cross shareholders on corporate cash holdings using a sample of Chinese A-share listed companies from 2007 to 2024. Regression results reveal that cross shareholders can increase corporate cash holdings and the potential channels of this increase are associated with reductions in overinvestment and improvements in total asset turnover. Heterogeneity tests indicate that the effect of cross shareholders is more pronounced under conditions of higher economic policy uncertainty, higher industry competition intensity, lower equity concentration and less analyst attention. Economic consequence tests reveal that cross shareholders not only increase corporate cash holdings but also enhance the positive effect of corporate cash holdings on total factor productivity. In summary, cross shareholders improve corporate cash management and boost operational efficiency.

Three implications can be drawn from the above findings. First, for cross shareholders themselves, they should fully leverage their positive role to enhance corporate governance and business performance. During the investment process, they should avoid passive “free-riding” behavior and instead actively exercise shareholder rights, participate in corporate operations and management, and achieve a win-win outcome for both corporate value and their own investment portfolios. Investors who are not yet cross shareholders may also consider diversifying their portfolio across multiple points within the same industry. This approach allows them to benefit from the advantages of cross shareholders and harvest substantial investment returns. Second, enterprises should strengthen communication and collaboration with cross shareholders, actively seek their advice, and fully integrate them into business decision making. By leveraging the cross shareholders’ equity advantages, companies can enhance overall performance and ultimately improve operational efficiency. Companies without cross shareholders, particularly those facing high external uncertainty, high industry competition intensity or weak governance mechanisms, should seriously consider introducing cross shareholders as an external catalyst to elevate their governance capabilities. Third, regulatory authorities should establish robust legislative oversight mechanisms for cross shareholders. On one hand, regulations governing cross shareholder must be refined, not only to design favorable policies encouraging high-quality cross shareholders to invest, but also to establish penalties for violations, thereby decreasing the cost of anti-competitive collusion by cross shareholders and eliminating the possibility of collusion at the policy level. On the other hand, authorities should strengthen oversight on the behavior of companies with cross shareholders to prevent market-disrupting activities such as insider trading and market manipulation. They must regulate related-party transactions among enterprises held by cross shareholders to ensure these shareholders do not undermine market fairness.

This paper has some limitations that could be further explored in future research. First, in terms of model specification, this paper employs industry and year fixed effects rather than firm fixed effects. This is primarily because the core explanatory variable *Cross* is constructed based on the same-industry criterion—it measures the number of shareholders that hold shares in at least two firms within the same industry. As such, the variation in *Cross* is primarily driven by the between-firm difference rather than the within-firm temporal fluctuations. Employing firm fixed effects would absorb the between-firm variation essential for identification, making it difficult to precisely estimate the effect of *Cross*. Nevertheless, we acknowledge that given the panel nature of the dataset, the results may be affected by unobserved firm-specific heterogeneity. Future research could explore higher-quality data or quasi-experimental designs to address this issue more rigorously. Second, the paper assumes cross shareholders are homogeneous, neglecting the differences among cross shareholders in voting power, investment objectives or the centrality of shareholders within the ownership network. Such method may be subject to measurement error, affecting the interpretation of the results. To address this limitation and to better capture the heterogeneity of cross shareholder influence, future studies are encouraged to develop more refined measures that can better capture the differences in voting power, investment objectives and network centrality. Thirdly, the mechanism analyses are indicative rather than strictly causal because potential simultaneity among investment, asset turnover, and cash holdings, as well as possible omitted variables may preclude us from making strong causal claims. Future research could employ methods such as quasi-experiments to establish strong causal relationships. Fourthly, this paper provides direct evidence that the presence of cross shareholders has a positive impact on cash holdings, but it does not directly address the effect of cross shareholders on the efficiency of corporate cash management. Future research could further explore this aspect.

## Supporting information

S1 Appendix Variable definitions. Detailed definitions of all variables used in this study.
(DOCX)

S2 Appendix Other robustness checks. Additional robustness checks using the Tobit model and firm fixed effects model to enhance the robustness of our findings.
(DOCX)

S1 File Stata source code. Stata code used to reproduce the results reported in this study.
(DO)

S1 Data Stata source data. Source data in Stata format used to reproduce the results reported in this study.
(DTA)

S2 Data Excel source data. Source data in Excel format, providing readers with a quick overview of the data used in this study.
(XLSX)
