# Supplementary material for: Cross shareholders and corporate cash holdings: Empirical evidence from Chinese listed companies
Source: PLoS One. 2026 Oct 26;21(10):e0338657. doi: 10.1371/journal.pone.0338657 (PMC13704850; doi:10.1371/journal.pone.0338657)
Supplement: Supplementary file 1 [file pone.0338657.s001.docx]

**S1 Appendix. Variable definitions**

| **Variable** | **Definition** |
| --- | --- |
| ***Measures of firm cash holding*** | |
| *Cash* | Cash and cash equivalents divided by total assets |
| ***Measures of cross shareholders*** | |
| *Cross* | The quantity of cross shareholders. Following He and Huang [3] and Chen et al. [11], we measure the quantity of cross shareholders based on these steps. Specifically, for each quarter in the 2007-2024 sample period, we extract Top 10 holdings and keep the data if a holding exceeds 5% of the outstanding shares. We define a shareholder as cross shareholder if he/she/it simultaneously holds more than one company in the same industry. Industry classification is based on the industry categorization of the Association of Listed Companies, with manufacturing subdivided into secondary codes and non-manufacturing enterprises subdivided into primary codes. We then calculate the annual average of the number of cross shareholders at the quarterly level, and take the natural logarithm of the sum of average data plus 1. |
| ***Control variables*** | |
| *Size* | Natural logarithm of total assets at the end of period |
| *Lev* | Total liabilities / Total assets at the end of period |
| *Top1* | Largest shareholder ownership |
| *Board* | Natural logarithm of the number of directors |
| *TMTpay* | Natural logarithm of the largest three managements’ salary |
| *ROA* | Net profit / Total assets at the end of period |
| *Growth* | (Current-Year Revenue / Prior-Year Revenue) - 1 |
| *Quick* | (Current assets – inventory) / Current liabilities at the end of period |
| *FC* | The absolute value of the SA Index; the higher the value, the more severe the financing constraint |
| *Nwc* | Net working capital / Net assets at the end of year |
| *Div* | Dummy with value one if cash dividends were distributed that year, zero otherwise |
| *Industry* | Industry dummies based on industry categorization of the Association of Listed Companies |
| *Year* | Year dummies from 2007 to 2024 |
| ***Other variables*** | |
| *Cross_IV* | The inverse of the weighted average distance of each company from the two major financial centers, Shanghai and Shenzhen, multiplied by the company’s listing age (lagged by one period) |
| *Finance* | The ratio of regional financial institution loan balances to regional GDP |
| *Age* | Firm listing age (lagged by one period) |
| *Cross_Dummy* | Dummy with value one if the firm-years observation has at least one cross shareholder, zero otherwise |
| *ROE* | Net profit / equity at the end of period |
| *Netprofit* | Net profit / Revenue |
| *Province* | Province dummies for provinces in China |
| *Cross_Share* | The total ownership of all cross shareholders in certain firm in certain year |
| *Cash1* | *Cash* minus the industry-year mean of *Cash* |
| *Cash2* | Cash and cash equivalents divided by total assets excluding cash and cash equivalents |
| *Overinv* | Referring Biddle et al. model, if the residual is larger than zero, *Overinv* equals to the residual, otherwise *Overinv* equals to zero |
| *ATO* | Total asset turnover ratio |
| *Epu* | According to the method used by Baker et al. [32], first calculates the monthly economic policy uncertainty index, then finds its annual average, and finally takes the natural logarithm of the annual average |
| *HHI* | Herfindahl-Hirschman Index, calculated as follows: first, the proportion of a single company's main business revenue relative to the total main business revenue of all companies in its industry is calculated; then sum the squared proportion by industry |
| *Share_HHI* | Calculated by squaring the shareholding ratios of the top 10 shareholders and summing the results |
| *Analyst* | Natural logarithm of the sum of analysts covering the same listed company in a year and 1 |
| *TFP* | Total factor productivity calculated by LP method |
