# Supplementary material for: Cross shareholders and corporate cash holdings: Empirical evidence from Chinese listed companies
Source: PLoS One. 2026 Oct 26;21(10):e0338657. doi: 10.1371/journal.pone.0338657 (PMC13704850; doi:10.1371/journal.pone.0338657)
Supplement: Supplementary file 2 [file pone.0338657.s002.docx]

**S2 Appendix. Other robustness checks**

Table 15 reports the marginal effects (dy/dx) from the Tobit estimation. In the Tobit estimation, the marginal effect of the core explanatory variable, *Cross*, is 0.011 and is statistically significant at the 1% level. This result is consistent with our baseline OLS estimates, providing further support for the conclusion that cross shareholders increase corporate cash holdings.

**Table 15. Results of Tobit model**

| Variable | (1) |
| --- | --- |
|  | *Cash* |
| *Cross* | 0.011*** |
|  | (3.57) |
| Controls | YES |
| Industry FE | YES |
| Year FE | YES |
| *N* | 45213 |

Note: The table reports marginal effects (dy/dx) on *Cash*. Robust t-values clustered at the firm level are reported in parentheses, ***, ** and * indicate statistical significance at 1%, 5% and 10% levels respectively (two-tailed).

Table 16 reports the results when using the firm fixed effects model. Under the firm fixed effects specification, the coefficient on *Cross* remains directionally consistent with the baseline model but is no longer statistically significant. We attribute this result primarily to the construction of *Cross* and the nature of its variation. Specifically, *Cross* is constructed based on the same‑industry criterion—it measures the number of shareholders that hold shares in at least two firms within the same industry. As such, it inherently captures industry‑level equity network structures rather than firm‑specific attributes. In other words, the identification of our core mechanism relies more on cross‑sectional differences in cross‑ownership structures across firms within the same industry than on short‑term temporal fluctuations within a given firm.

To further understand the variation structure of *Cross*, we decompose its variance. The total variance is 0.249, with a within‑firm variance of 0.143 and a between‑firm variance of 0.206. Notably, the between‑firm variance substantially exceeds the within‑firm variance, indicating that the variation in *Cross* is predominantly driven by cross‑sectional differences across firms rather than by temporal fluctuations within firms.

When firm fixed effects are applied, the between‑firm variation in *Cross* is largely absorbed, leaving only within‑firm variation for identification. This undermines the primary source of variation in *Cross* and makes it difficult to estimate its effect precisely. By contrast, industry fixed effects preserve the between‑firm variation in *Cross* while controlling for time‑invariant industry‑level confounders, which is more congruent with both the construction of *Cross* and its variation structure. Moreover, we have included a comprehensive set of firm‑level control variables in our baseline specification to further mitigate potential omitted variable bias. Taken together, we believe that our baseline specification with industry fixed effects is appropriate given the construction of *Cross* and the predominance of between‑firm variation in its overall variance. Nevertheless, we acknowledge that the insignificance of *Cross* under firm fixed effects suggests that our baseline association remains sensitive to unobserved firm‑specific heterogeneity. Accordingly, while our core conclusions are supported by a range of robustness checks, we interpret them with due caution.

**Table 16 Results of firm fixed effects**

| Variable | （1） |
| --- | --- |
|  | *Cash* |
| *Cross* | 0.005 |
|  | (1.26) |
| *Controls* | YES |
| Firm FE | YES |
| Year FE | YES |
| Constant | 0.343*** |
|  | (4.78) |
| *N* | 45213 |
| Adjusted R^2^ | 0.650 |

Note: Robust t-values adjusted by heteroscedastic and firm-level cluster standard errors are below the regression coefficients in parentheses, ***, ** and * indicate statistical significance at 1%, 5% and 10% levels respectively (two-tailed).
